# A Study on the Influential Factors of Stress Corrosion Cracking in C110 Casing Pipe

**DOI:** 10.3390/ma15030801

**Published:** 2022-01-21

**Authors:** Zhe Wang, Pingquan Wang, Dezhi Zeng, Taihe Shi, Wenliang Deng

**Affiliations:** 1 State Key Laboratory of Oil & Gas Reservoir Geology and Exploitation, Southwest Petroleum University, Chengdu 610500, China; zengdezhi1980@163.com (D.Z.); taiheshi@126.com (T.S.); 2 China Petroleum Pipeline Engineering Co., Ltd., Langfang 065000, China; dengwenliang2022@163.com

**Keywords:** C110 casing pipe, stress corrosion cracking, fracture analysis, pitting pits, hydrogen-induced cracking

## Abstract

In this paper, we analyze the potential factors affecting the hydrogen sulfide type of stress corrosion cracking in C110 casing pipes. In order to further study these cracking factors, the methods of material property testing, scanning electron microscopy, XRD, TEM, and 3D ultra-depth-of-field were applied in the experiments. Besides that, an HTHP autoclave was independently designed by the laboratory to simulate the actual corrosion environment, and the potential factors affecting the stress corrosion cracking of C110 casing pipes were determined. The test results showed that the chemical composition, metallographic structure, hardness, and non-metallic inclusions of the two types of C110 casing pipes were all qualified. In fact, there remains a risk of stress corrosion cracking when the two kinds of C110 casing pipes serve under long-term field-working conditions. It is considered in this paper that the precipitates on the material surface, stress damage, and pitting corrosion are all critical factors affecting the stress corrosion cracking of casing pipes.

## 1. Introduction

In the oil and natural gas extraction process, casing pipe failures caused by corrosion lead to casing pipe fractures from time to time in the procedure of on-site work [1]. With the rapid increase in demand for oil and gas resources, acid oil and gas fields have been widely exploited [2]. As a result, the casing pipes have been jointly affected by temperature, pressure, stress, acid corrosion medium, and other factors during service, which poses a certain level of fracture risk and hidden danger [3]. According to some studies in the literature [4,5,6,7], the uniform corrosion rate of casing pipe in a H_2_S-CO_2_ corrosion environment is not the major consideration in material selection and design, but its fracture resistance in a H_2_S environment turns out to be the key, and stress corrosion cracking is regarded as the leading factor in casing pipe fracture. Therefore, this problem deserves more attention.

Many experts have conducted in-depth studies on the corrosion behavior of carbon steel and low-alloy steel in a H_2_S-CO_2_ environment [8,9,10,11,12]. Petrov [13], Shi [14], Henthorne [15], and Wen [16] studied the stress corrosion cracking of different grades of steel in acidic H_2_S-CO_2_-Cl^−^ solution, and they obtained the relationships between the corrosion rate and external corrosion parameters (solution component concentration, temperature, and tensile stress) as well as internal corrosion parameters (Cr, Ni, and Mo doping levels and conditional yield stress of steel), which assisted users in evaluating the corrosion behavior and the steel application possibilities in corrosive environments. Wang [17] and Zhao [18] researched the corrosion rate and sulfide stress corrosion cracking (SSCC) phenomenon of P110SS casing steel under working conditions with a high content of H_2_S-CO_2_, and they found that increases in H_2_S partial pressure, CO_2_ partial pressure, and temperature were followed by a decrease in SSCC sensitivity, with temperature playing a leading role, while SSCC sensitivity became the highest under wellhead conditions. Zhao [19], Zhang [20], Qiu [21], and others conducted high-temperature, high-pressure immersion tests to discover that uniform corrosion mainly occurred on the pipe surface when H_2_S pressure was low, but with increasing H_2_S pressure, pitting corrosion gradually emerged, resulting in an increase in the cracking sensitivity of the pipe material. Xue [22] and Lian [23] performed performance evaluation test studies simulating resistance to stress corrosion cracking (SCC), and they concluded that the NACE C-ring test method could reflect the influence of pipe surface status on its SCC resisting performance. It was recommended to apply the C method to assess the SCC resisting performance of stainless steel oil pipes. However, no evaluation method has been proposed for C110 sulfur-resistant steel.

Through the above research, experts acquired a deep understanding and additional research methods to examine the mechanism of stress corrosion cracking in casing pipes in a H_2_S-CO_2_ environment. In addition, it can be seen that the research on H_2_S-CO_2_ corrosion of steel mainly focused on indoor simulation experiments. However, the circumstances of pipes in a pitshaft are so complex that it has been rather difficult to completely simulate the pitshaft circumstances in laboratory tests. Zhu [24], and Lei [25] analyzed the failure causes of Super 13Cr Steel in high-temperature, high-pressure wells of sour gas, attributing the failures to stress corrosion cracking, and they pointed out that the mechanism of stress corrosion cracking is fracture of the corrosion product film. However, they did not analyze the influential factors of film fracture from stress corrosion cracking of Super 13Cr Steel in high-temperature, high-pressure wells of sour gas. Deng [26] et al. analyzed failed casing pipe samples and concluded that the major reason for C110 sample failure was the low tempering temperature and the large residual stress in the steel. However, the downhole service environment and working conditions of casing pipes are so complicated that many other factors deserve attention, on top of the influence of the manufacturing technique on pipe material. Therefore, it is necessary to further study the influential factors causing stress corrosion cracking of C110 casing pipes under actual working conditions.

Through the connection between laboratory research and operating conditions, in this study, we proposed to carry out constant-load tests in a high-temperature, high-pressure environment under conditions imitating the actual working environment, in order to better simulate the real state of a casing pipe serving in a pitshaft. By studying the mechanical properties and tensile fracture morphology of two kinds of tensile samples with constant load under high-temperature, high-pressure circumstances, many factors of stress corrosion cracking in casing pipes under acidic conditions were analyzed, with the purpose of providing a certain reference basis for selecting the proper oil casing pipe from the perspective of stress corrosion cracking.

## 2. Experiments

### 2.1. Materials

For this paper, we selected two kinds of C110ksi casing pipes commonly used in oil fields as the research objects, named C110-1 and C110-2. Their chemical compositions are shown individually in Table 1.

The samples were processed according to the φ = 6.35 mm standard in NACE TM0177-2005, while the sample size, accuracy requirements, and tensile test parameters were all in strict accordance with the requirements of GB/T 228-2002; the yield strength, tensile strength, and elongation of the two materials were then obtained by stretching them on an MTS810 testing machine (Metex Industrial Systems Co., Ltd., Shenzhen Branch, China), as recorded in Table 2.

### 2.2. DCB Experiments

#### 2.2.1. Experimental Equipment

**Figure 1:**
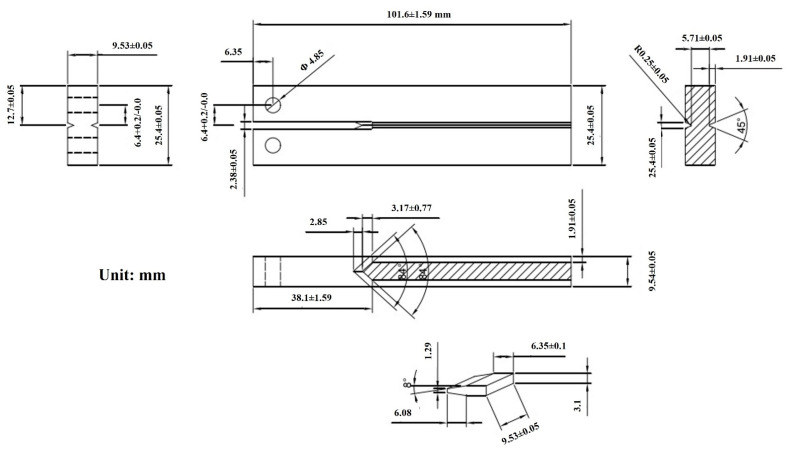
Schematic illustrations of specimen size (DCB).

According to the regulations of NACE TM0177-2005 method D, i.e., the Double Cantilever Beam (DCB) method, we made samples and load wedge blocks (Figure 1) from the C110-1 and C110-2 casing pipe materials, then conducted experiments in a standard A solution of corrosion medium.

The experimental medium was composed of 5.0% (wt.%) sodium chloride and 0.5% glacial acetic acid both dissolved in distilled water, after which hydrogen sulfide was introduced into the experimental medium solution to reach saturation. The initial pH value was required to be within the range of 2.6 to 2.8.

**Figure 2:**
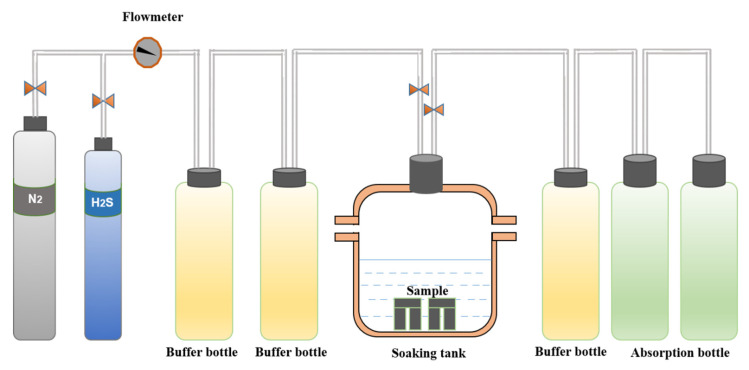
Schematic diagram of the experimental set-up (DCB).

The testing device adopted in this experiment is shown in Figure 2 and was designed in strict accordance with the D method in NACE TM0177-2005 [27].

**Figure 3:**
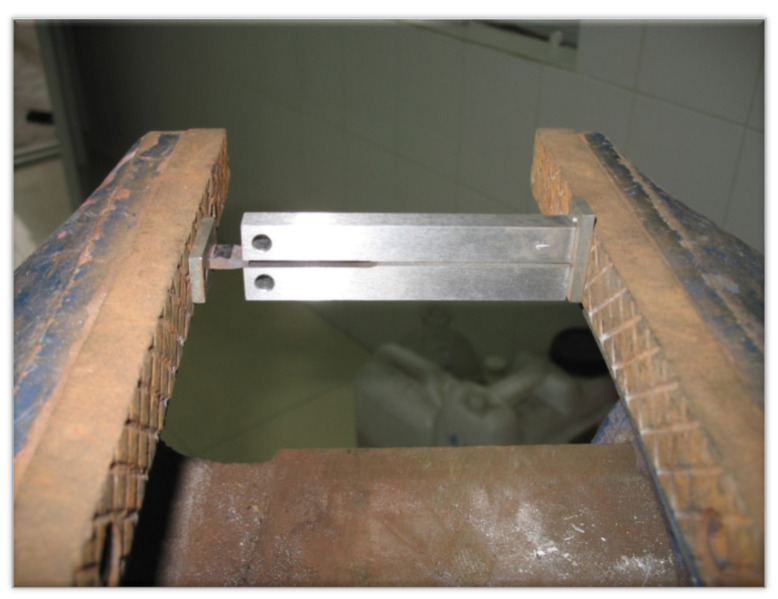
Experimental DCB loading device.

In order to carry out the test in strict accordance with the standard, the small wedge block loading device was nipped with the vice of the bench clamp seat (Figure 3) to ensure that they were all clamped in and aligned at the fracture of the test specimen.

The C110-1 and C110-2 selected in the experiment are both 110 ksi sulfur-resistant steels. According to the provisions on cantilever displacement (Table 3) in ISO 11960 standard, their value should be around 0.38–0.64 mm, and the dimension of wedge thickness D should be within the range 2.38 + 2δ. Considering the deformation of the material contact surface during loading, an area with large thickness should be selected, and then the initial spacing of loading holes on the test specimen should be measured to determine the actual value of cantilever displacement.

#### 2.2.2. Experimental Process

Firstly, the composition and mechanical properties of non-corroded C110-1 and C110-2 were analyzed. Secondly, each group of samples was put into the corresponding position of the soaking tank before adding the prepared experimental solution. Thirdly, the soaking tank was sealed, and every liter of solution was fully deoxidized with N_2_ for 1 h. After the deoxidization, there was a concern that the experimental solution would become turbid (opaque) as an obvious phenomenon of oxygen pollution when H_2_S gas was introduced into the soaking tank, which would prevent the polluted solution from being applied in the experiment. For this reason, the test pieces were taken out and cleaned, while the solution was prepared again according to the procedure and the oxygen removed. Finally, the experimental solution was filled with H_2_S at a minimum flow rate of 100 mL/min until saturation. During the experiment, the continuous flow of H_2_S between the soaking tank and the outlet filter was kept at a low flow rate of 10 bubbles per minute, in order to maintain the H_2_S concentration and small positive pressure in the experimental solution, with the purpose of preventing air from entering the experimental container from small leaks. After completing the above steps, the experiment was set to start. The test parameters were set as follows: entire experimental duration, 14 days; temperature, 24 ± 3 °C.

After the experiment, the corroded DCB sample was taken out of the soaking tank, and the corrosion products were removed from the surface of the sample by distilled water before drying. Subsequently, tensile testing was conducted with the dried sample on the tensile testing machine to obtain the balanced wedge load *P*. Since it was difficult to clearly observe the crack when the sample was taken out, and the length of the crack could not be determined, the sample was then condensed with liquid nitrogen and cleaved by quick freezing. After that, the macro morphology, mechanical properties, and tensile fracture morphology of the cleaved corrosion samples were analyzed thoroughly.

#### 2.2.3. Fracture Morphology and Microstructure Analysis

As a matter of fact, the specimens will be affected by the corrosive medium during the cracking. Therefore, it is necessary to test the microstructure of samples before and after corrosion, in order to study the stress corrosion cracking mechanism of the experimental materials. In detail, the fracture morphology of the DCB samples was observed by scanning electron microscopy (TESCAN ORSAY HOLDING, a.s., Brno, The Czech Republic) to clarify the fracture process of the sample. A TEM (Beijing Opton Optical Technology Co., Ltd., Beijing, China) was also applied to observe the submicroscopic structures of the two kinds of casing pipe steel samples, and their SSC resistance was analyzed and compared as well.

### 2.3. Constant-Load Experiment

The experimental conditions of NACE standard A solution are very aggressive. Through a large number of on-site practice runs, it is already proved that the material unqualified in standard A solution does not crack during long-term downhole operation. The actual environment is not as harsh as the experimental environment of standard A solution, and in the real environment, the annulus protection fluid in an effectively protective state can not only inhibit the electrochemical corrosion of materials, but also slow down the stress corrosion of casing pipes. Therefore, it is imperative to evaluate the applicability of environmental cracking of the two kinds of C110 casing pipes by the method of high-temperature, high-pressure constant load under conditions simulating the actual working environment.

#### 2.3.1. Experimental Equipment

**Figure 4:**
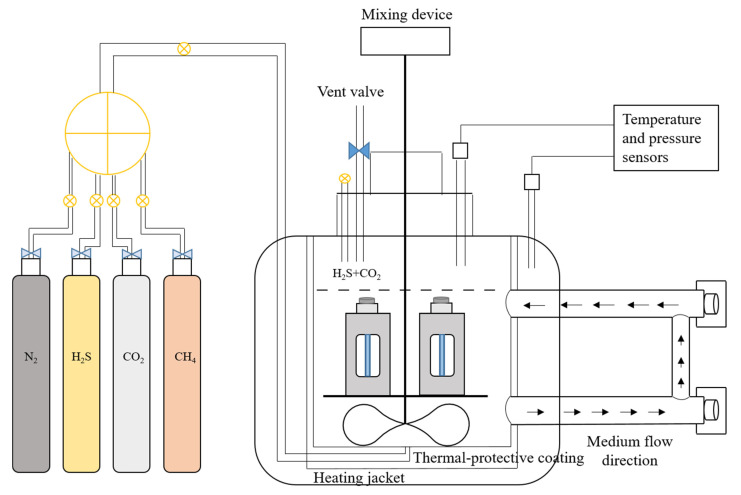
The stress corrosion device with constant load.

**Figure 5:**
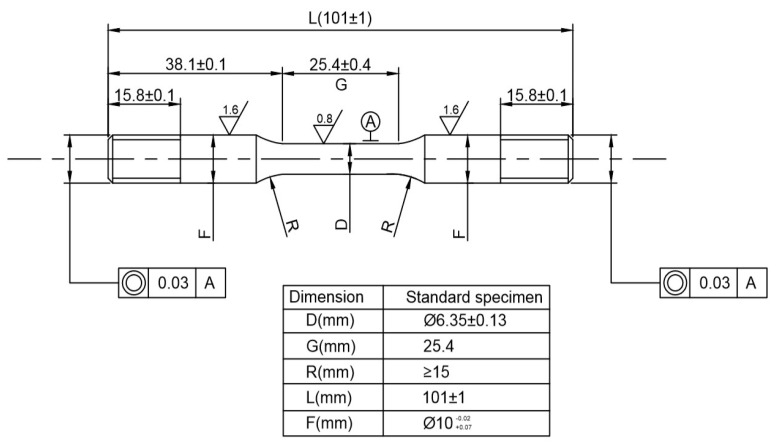
Dimensions of specimens for constant-load tests.

In order to study the hydrogen damage characteristics of C110-1 and C110-2 under constant load, a dynamic circulating multiphase flow experimental autoclave at 70 MPa and 200 °C was applied by the research group, in addition to the stress corrosion device with constant load and plate fixture designed by Hastelloy C276 (Figure 4). The loaded specimen of φ 6.35 mm was finally processed according to the NACE TM0177-2005 standard (Figure 5).

**Figure 6:**
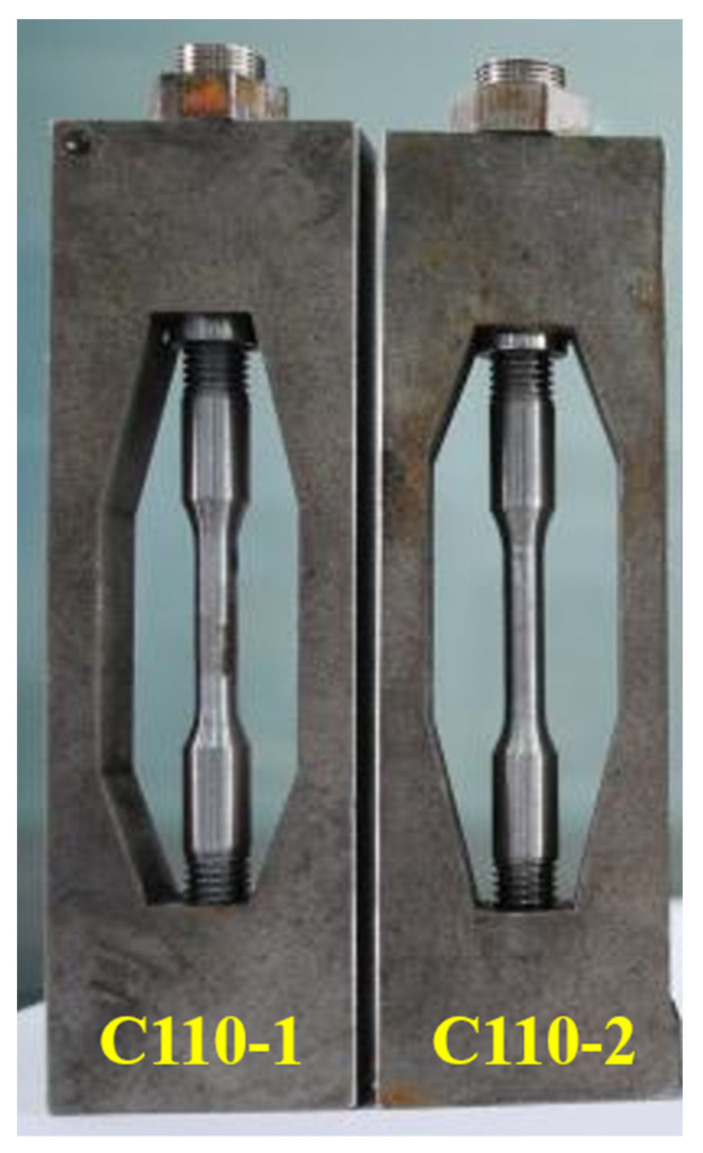
The constant-load device for plate fixture.

The sample and the fixture were assembled before preloading them (Figure 5 and Figure 6) on a tensile testing machine. Hou Duo, Shi Taihe, and others [28] already verified the reliability of the loading mode and fixture.

#### 2.3.2. Experimental Process

Firstly, the corrosive medium was determined according to the on-site working conditions; then, the simulated formation water was prepared and was fully deoxidized. The ion contents of the water and the dosages of prepared reagents are shown in Table 4 and Table 5. Secondly, the autoclave was cleaned and a pressure test was conducted to ensure good sealing, after which the temperature of the autoclave was raised to that of the experimental requirements. Thirdly, the loaded fixture was put into the autoclave with the simulated formation water after deoxidization, then the autoclave was processed at high temperature and high pressure with another deoxidization (removal of oxygen from the experimental environment during operation). Finally, H_2_S, CO_2_, and N_2_ were introduced successively to meet the pressure requirements of the experimental conditions, while the mixer was turned on to continue the experiment. The test parameters were set as follows: partial pressure of CO_2_, 2 MPa; partial pressure of H_2_S, 1 MPa; total pressure, 10 MPa; entire experimental duration, 168 h; and temperature, 60 °C.

After reaching the end of the experimental time, the autoclave was cooled down and opened to take out the experimental fixture, then tensile fracture testing was conducted on the MTS testing machine, and the stress–strain curves were recorded. The mechanical properties and tensile fracture morphology of the corrosion samples were both analyzed specifically.

#### 2.3.3. Mechanical Property Testing

The sample was fixed into the clamping device on the slow tensile MTS testing machine and was loaded slowly. The tensile rate of the testing machine set to 1 mm/min (strain rate 6.6 × 10^−4^ s^−1^), and the mechanical property test samples were run at room temperature (23 °C) after the corrosion test.

#### 2.3.4. Fracture Morphology and Surface Damage

In order to study the stress corrosion damage to materials, it is essential to observe the microstructure of the fracture surface after the corrosion test by scanning electron microscopy. In addition, the corrosion morphology of the sample surface was analyzed and measured by ultra-depth-of-field 3D microscopy (Keyence Corporation, Osaka, Japan) after the stress corrosion.

## 3. Results and Discussion

### 3.1. Analysis of Metallographic Structure, Microstructure, and Submicroscopic Structure

**Figure 7:**
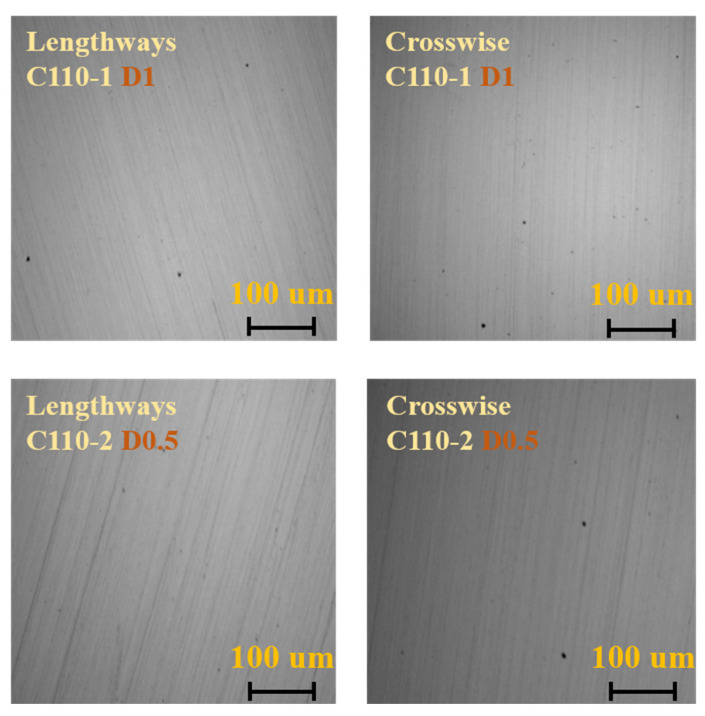
Non-metallic inclusions in C110-1 and C110-2.

**Figure 8:**
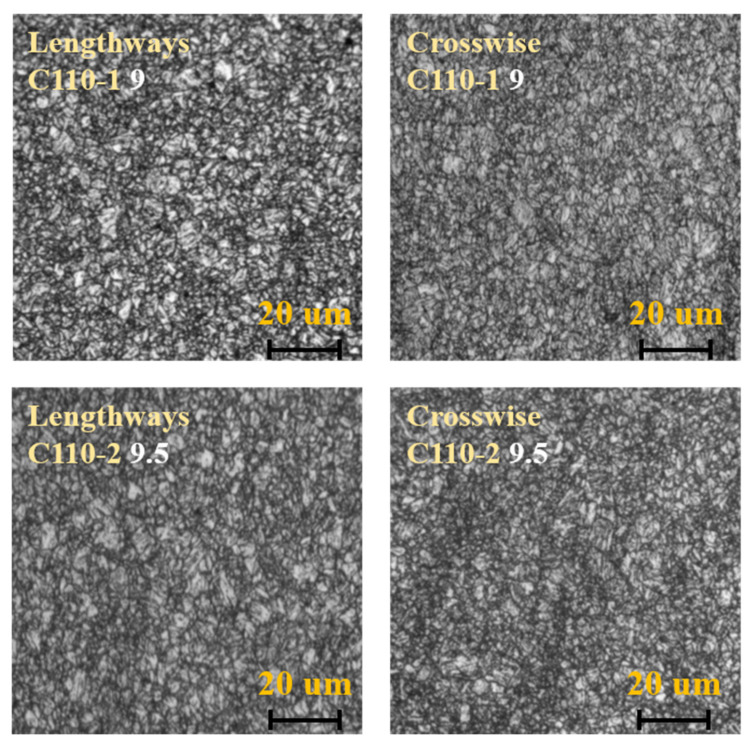
Grain size of C110-1 and C110-2.

**Figure 9:**
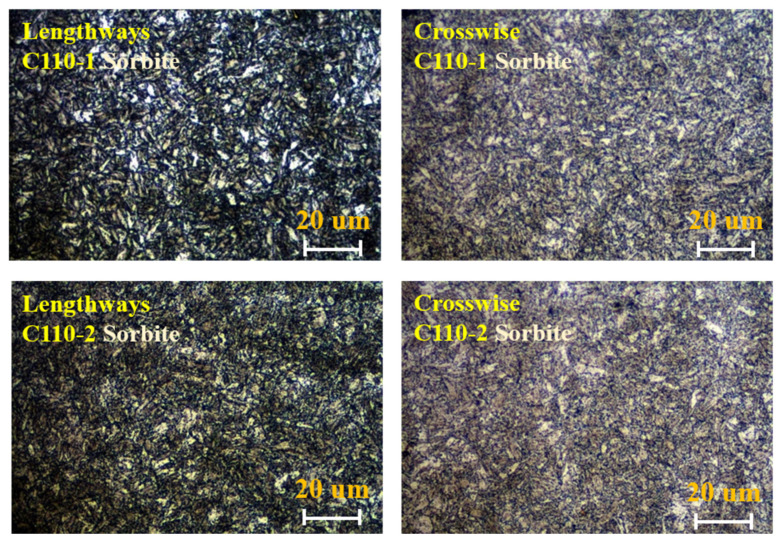
Metallographic structure of C110-1 and C110-2.

According to the ASTM E45-2005, ASTM E112-1996, and GB/T 13298-1991 standards, samples of 10 mm × 10 mm × 10 mm thickness were cut from C110-1 and C110-2 casing pipes for metallographic analysis, including non-metallic inclusions (ASTM E45-2005), grain size (ASTM E112-1996), and microstructure (GB/T 13298-1991), as shown in Figure 7, Figure 8 and Figure 9. The results showed that the C110-1 sample has ASTM D1 fine non-metallic inclusions, a grain size of ASTM 9, and a microstructure of tempered sorbite. The C110-2 sample has ASTM D0.5 coarse non-metallic inclusions, a grain size of ASTM 9.5, and a microstructure of tempered sorbite.

Through comparative analysis, it can be seen that firstly, the size of inclusions (non-metallic oxides) in C110-1 is larger than that in C110-2. The results showed that the non-metallic inclusions in the C110-1 sample were ASTM D1 fine, and the non-metallic inclusions in the C110-2 sample were ASTM D0.5 coarse. The inclusion zone is the stress concentration area of metal materials, the so-called fracture source inclusion. The fracture starts from the inclusion zone and occurs under the action of stress. Secondly, the grain size of C110-2 is smaller than that of C110-1. C110-1 was at the grain size ASTM 9 but C110-2 was at the grain size ASTM 9.5. Thirdly, in the microstructure of C110-2, the amount of retained austenite (white particles) is obviously small and uniformly distributed as fine particles. During the process of heat treatment, the distribution of residual austenite in steel has an important impact on the properties of the steel. The existence of a few residual austenite particles plays a certain role in improving the general strength and ductility of the steel. The reason for this is that the retained austenite among the martensite exists in a film shape, which can alleviate the stress concentration and prevent the generation and propagation of crack sources, improving the ductility of the steel. When the retained austenite in the steel exceeds a certain quantity, it plays a contradictory role in significantly lowering the hardness and wear resistance of the steel. Therefore, it can be inferred from the metallographic microstructure that domestic C110 is more prone to cracks and resultant fractures than SMC110, under the same stress conditions.

**Figure 10:**
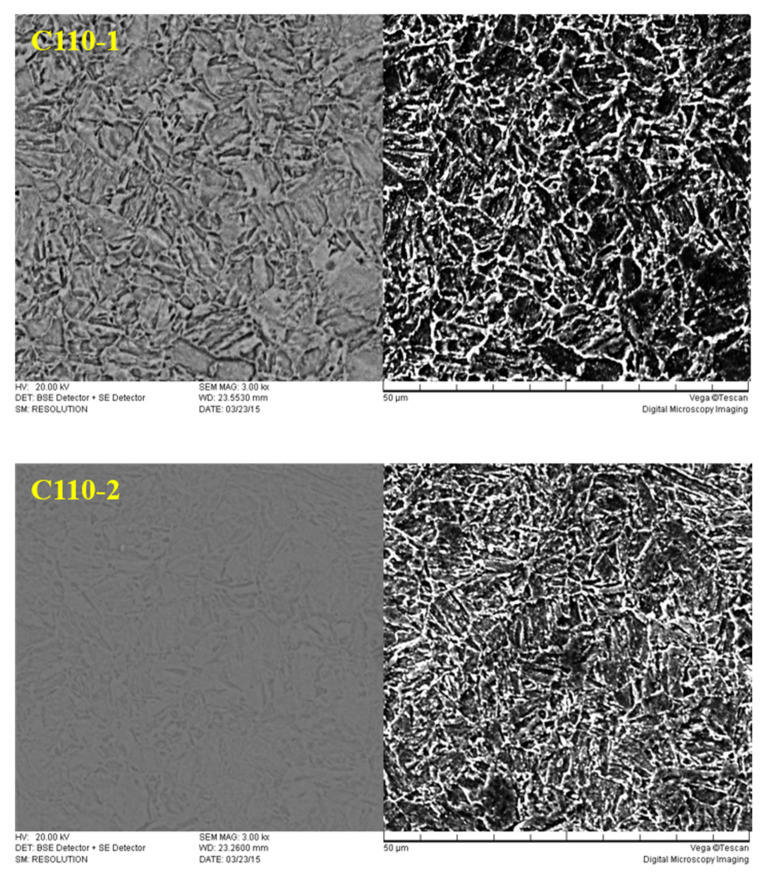
Microstructure of C110-1 and C110-2.

The microstructure and morphology of C110-1 and C110-2 samples were analyzed by SEM (scanning electron microscope), as shown in Figure 10.

Figure 10 shows the scanning organizations of secondary electrons and back-scattered electrons of C110-1 and C110-2 casing pipe materials. Both the heat treatment processes of the two steels include adjustments of quenching and high-temperature tempering, so their microstructures are both tempered sorbite. Tempered sorbite is composed of recrystallized ferrite and uniformly distributed coarse granular cementite. In C110-1 steel, ferrite has lost the acicular form of martensite formed in the original quenching process through re-crystallization and has become polygonal particles. At the same time, cementite aggregates and grows to form large carbides. Meanwhile, in C110-2 steel, ferrite also recrystallizes, but its carbide particles are smaller in contrast.

**Figure 11:**
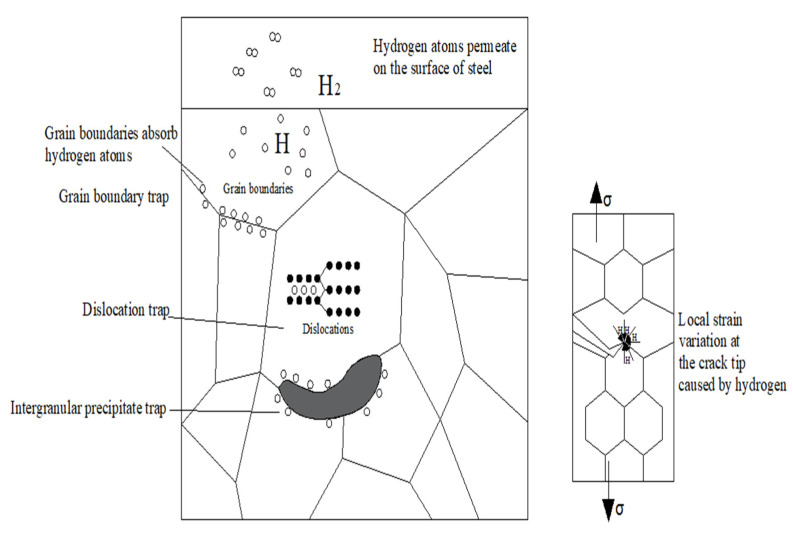
Hydrogen trap forms of steel.

The metal material is composed of many grains, and there are grain boundaries between them. Some studies [29] have shown that crystals grow in different forms due to the heat treatment technique and the addition of alloy elements in the process of nucleation and growth; as a consequence, there will be certain point defects such as vacancy, interstitial atoms, and replacement atoms between grain boundaries, causing grain boundary defects, also known as grain boundary traps. There are also vacancies in dislocations and intergranular precipitated phases, resulting in defects that have a certain impact on the mechanical properties of the material. The hydrogen trap form of steel is shown in Figure 11 [30].

**Figure 12:**
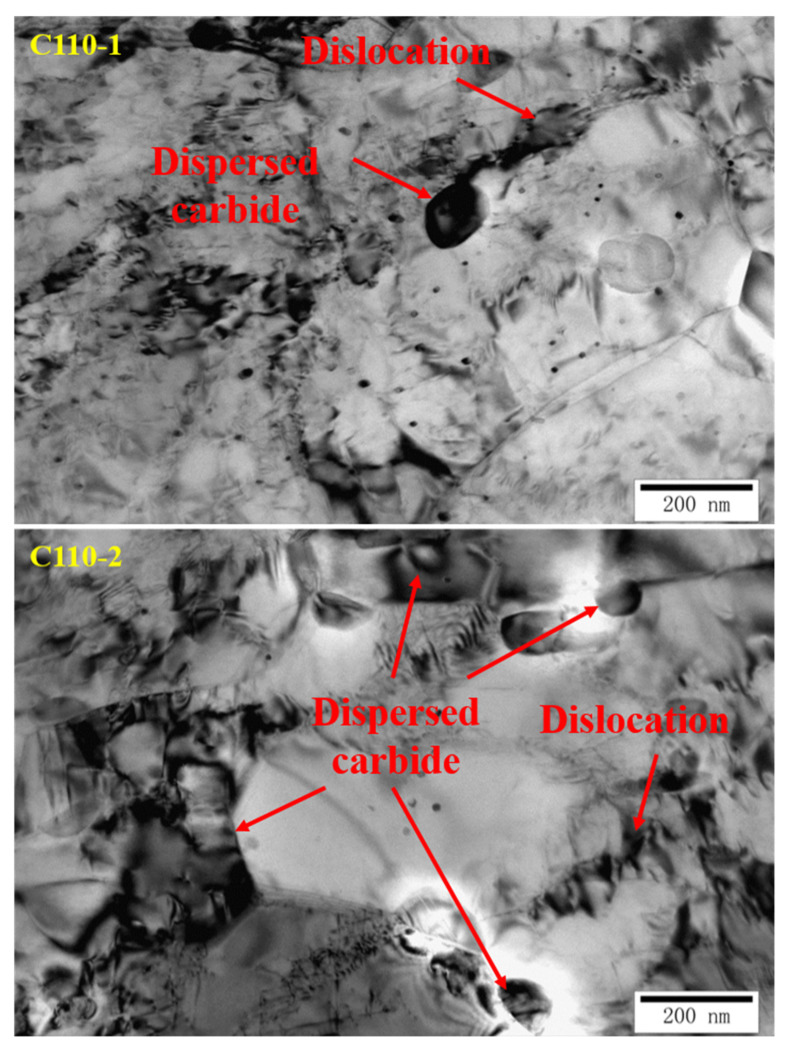
Submicroscopic structure of C110-1 and C110-2.

The submicroscopic structures of the C110-1 and C110-2 samples were observed by TEM and are shown in Figure 12. It can be seen that C110-1 and C110-2 basically have no banded pearlite, which is particularly sensitive to stress corrosion cracking generation and propagation, while all the grain sizes are relatively small. Moreover, the hydrogen pressure caused by hydrogen infiltration is borne by more fine grains; it can also be seen that C110-2 contains fewer inclusions and is thus prone to neither causing hydrogen embrittlement nor producing microcracks. In the crack propagation stage, the grains are small and there are many grain boundaries, so microcracks will be hindered at the grain boundary and will be unable to expand and grow easily. C110-2 is mainly composed of fine acicular ferrite. According to the relevant literature [31,32,33,34] about “effective grain size”, these interlocking and intertwining fine bundles could effectively hinder further crack propagation.

**Figure 13:**
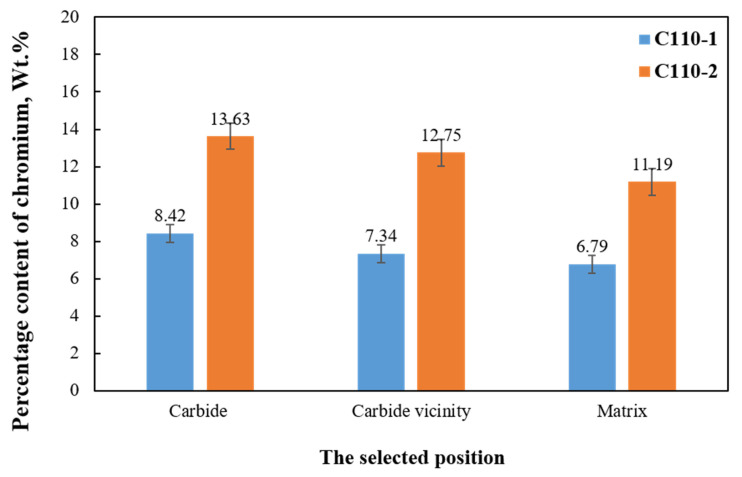
Comparison of Cr content.

In the H_2_S environment, hydrogen atoms enter the material, with one part distributed in the lattice gaps, and the other reacting with various reversible or irreversible hydrogen traps in the material. The carbides dispersed in the microstructure exist in the matrix in the form of hydrogen traps, providing many vacancies for the redistribution of infiltrated hydrogen atoms, which can effectively hinder the microregional hydrogen embrittlement caused by high hydrogen pressure in partial regions; this weakens the increase in partial hydrogen concentration caused by the fusion of infiltrated hydrogen and strong hydrogen traps in the material, and as a result, the possibility of crack generation is also reduced. Besides this, the dispersed carbide can improve the SSC resistance of the material as well. It can be seen from Figure 13 that there are more dispersed carbides and more uniform distributions in parts of C110-2 than in C110-1.

Three position points (matrix, carbide, and carbide vicinity) were selected from the surfaces of the two samples for EDS energy spectrum testing to obtain the Cr content (which varies greatly) in the corresponding elements, as shown in Figure 13. It can be seen that C110-2 steel precipitates more strong carbides—chromium carbides—during the tempering process, compared with C110-1 steel. Strong carbides can increase the diffusion activation energy of carbon within martensite and reduce the diffusion coefficient, slowing and delaying the decomposition of martensite. In other words, considering that C110-2 contains twice as much chromium, the amount of strong carbides is proportional to the Cr content.

Additionally, the dislocations in the material also have a great impact on the SSC resistance. Hydrogen can promote the proliferation of dislocations, activate dislocation initiation, and accelerate the formation of Persistent Slip Bands on the surface. Hydrogen forms an air mass around the dislocation, which can move along with the dislocation. Comparing the low dislocation density of C110-1 and the staggered entangled dislocation network of C110-2, C110-2 has lower dislocation mobility than C110-1, while the dispersed carbides also have an intense pinning effect on the dislocation. Therefore, the possibility of hydrogen embrittlement caused by dislocation movement is substantially reduced. To a certain extent, this kind of dislocation acts as a trap for infiltrated hydrogen atoms, which is beneficial to improving the SSC resistance of the material. As a result, C110-2 material, mainly composed of needle ferrite, has significantly improved SSC resistance due to its high-density dislocation inside and intense hydrogen traps from dispersed carbides.

### 3.2. Analysis of K_ISSC_ and Fracture Morphology in the DCB Experiment

To assess the fracture ductility, the critical stress strength coefficient of samples was determined according to Equation (1):

(1) $$K_{\mathrm{ISSC}}=\frac{Pa(2\sqrt{3}+2.38h/a){\left(B/Bn\right)}^{\frac{1}{\sqrt{3}}}}{Bh^{\frac{3}{2}}}$$

where *K*_ISSC_ is the critical stress intensity coefficient of SSC; *P* is the load of the balancing wedge, sourced from the actual value measured on the tensile testing machine; *a* is the crack length; *h* is the height of each cantilever; *B* is the thickness of the test piece; and *B_n_* is the thickness of the web.

**Figure 14:**
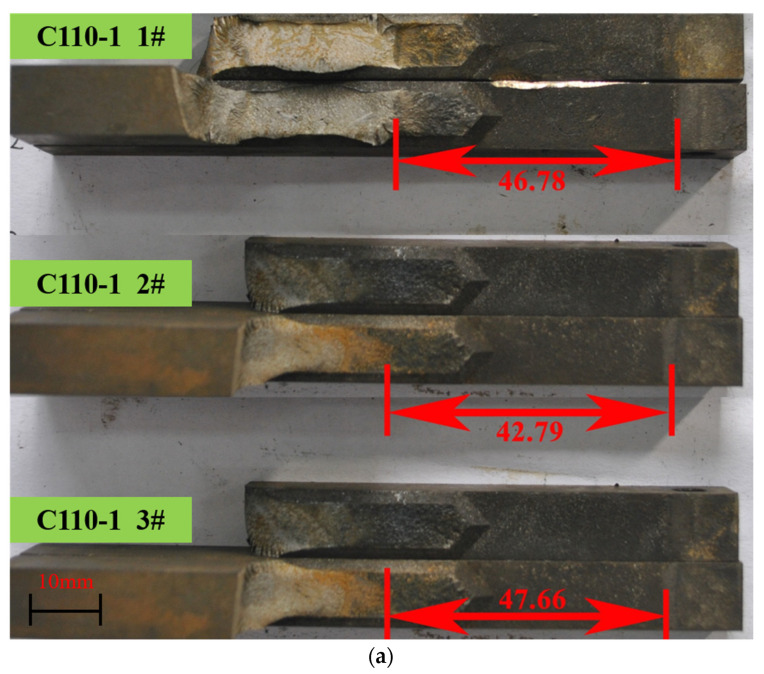

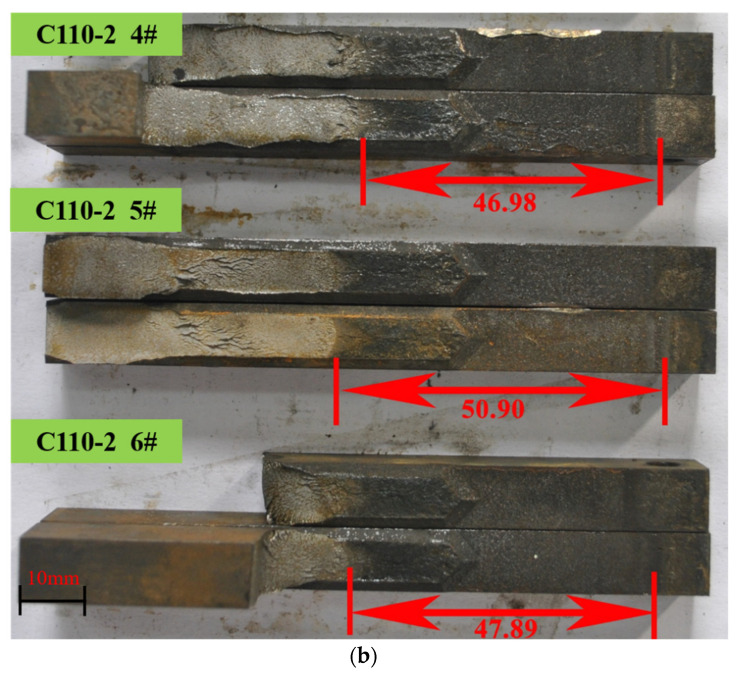
Photographs of samples after quench splitting by liquid nitrogen. (**a**) C110-1 01#, 02#, and 03#. (**b**) C110-2 04#, 05#, and 06#.

We measured the distance L from the slotted end of the test piece to the middle of the cracked end using a scale caliper. The crack length *a* was obtained by subtracting 6.35 mm from this distance, which is the length marked in red in Figure 14.

The test results of *K*_ISSC_ values indicating the environmental fracture ductility of the two samples are shown in Table 6; the *K_IC110-1_* value of C110-1 material was 27.16 MPa∙m^1/2^, and the *K_IC110-2_* value of C110-2 material was 28.19 MPa∙m^1/2^. The critical fracture ductility values of the C110-1 and C110-2 steels both meet the requirements of ISO 11960 (the *K*_ISSC_ values of the individual samples are both higher than 26 MPa∙m^1/2^). However, the C110-2 material shows weaker environmental cracking sensitivity under this circumstance, where the C110-1 steel shows stronger environmental cracking sensitivity and is more prone to cracking occurrence.

**Figure 15:**
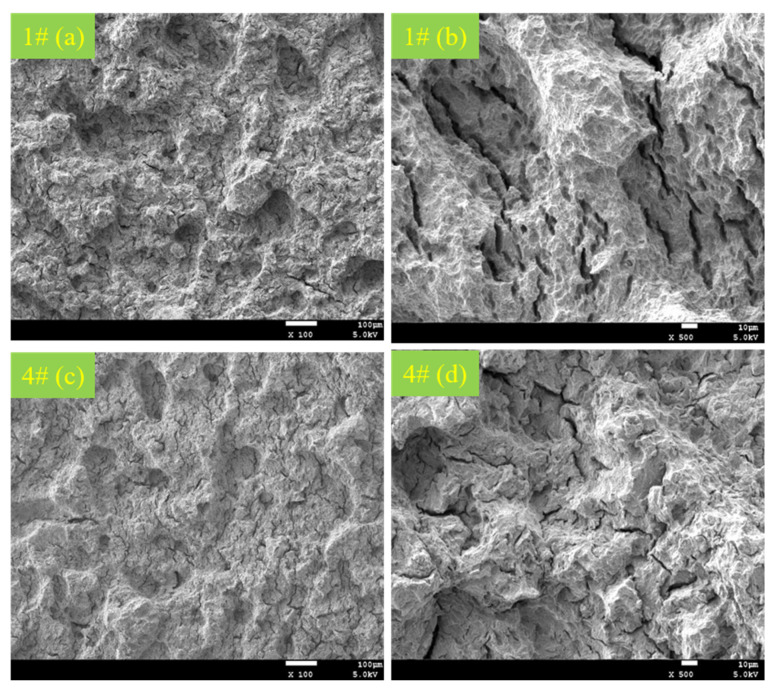
Microstructural morphology of ×100 (**a**,**c**) and ×500 (**b**,**d**) fractures after corrosion in samples of C110-1 (**a**,**b**) and C110-2 (**c**,**d**).

Two representative samples (1# and 4#) were selected for micromorphology analysis; the specific micromorphology of DCB fracture in these samples is shown in Figure 15 below.

It can be seen from Figure 15a,c that the fracture micromorphologies of C110-1 and C110-2 after the NACE D method test are not significantly different from each other; they are basically composed of dimples, ligaments, and shear steps at different heights, and both are classified as dimple ductile fracture. However, it can be seen from Figure 15b,d that the number and length of cracks on the fracture surface of the C110-1 sample are both greater than those of C110-2 under the same experimental conditions, indicating that the fracture ductility of C110-2 is better than that of C110-1, which is consistent with the previous experimental results (*K_IC110-1_* < *K_IC110-2_*).

In general, microcracks appeared in both the macro- and micromorphology of the two samples under the condition of saturated H_2_S in standard A solution, indicating the occurrence of stress corrosion cracking. However, these two kinds of casing pipes are suitable for on-site conditions in engineering applications and have been in service for over a decade. This is because standard A solution is the most severe environment and is not fully suitable for evaluating the on-site applications of these casing pipes.

### 3.3. Analysis of Stress Damage

**Figure 16:**
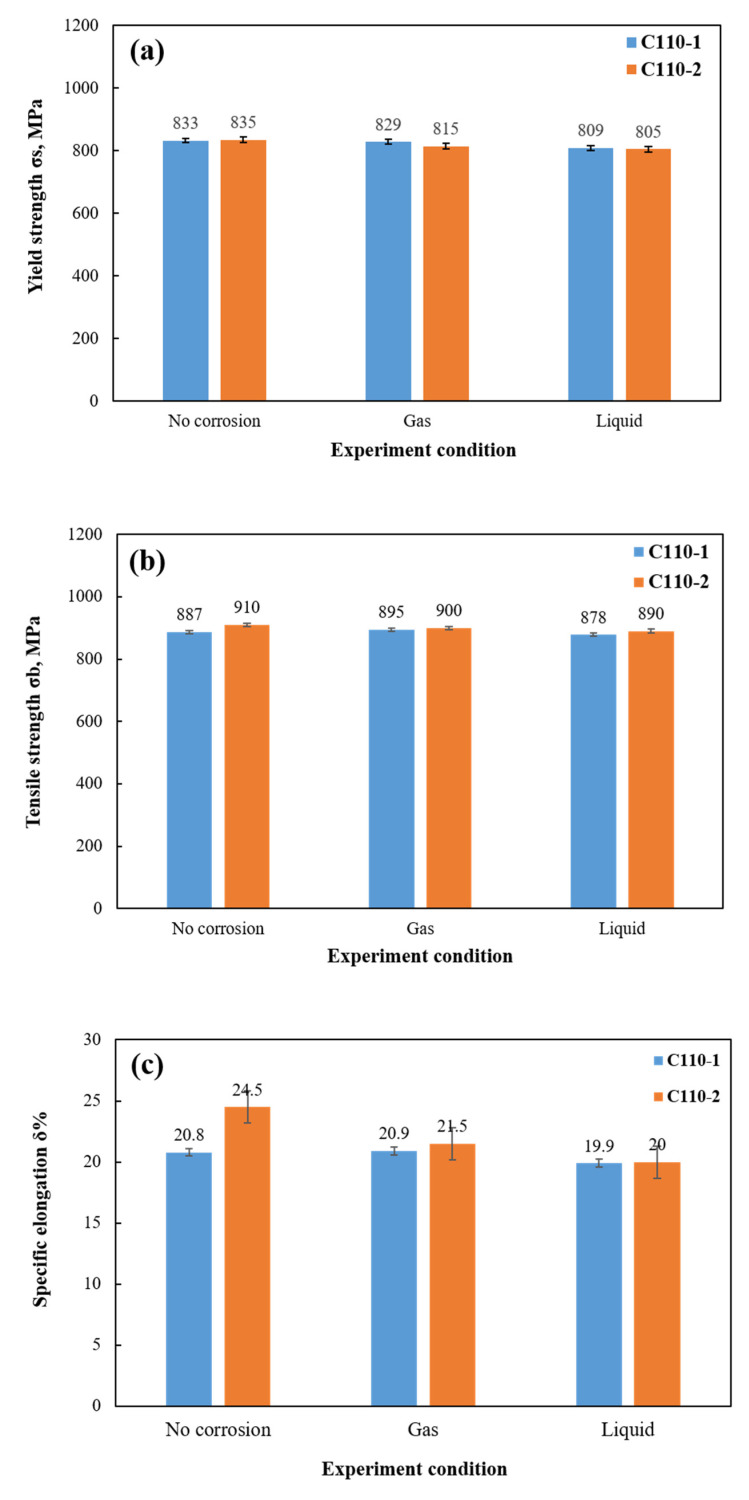
Comparison of mechanical properties: (**a**) Yield strength; (**b**) Tensile strength; (**c**) Specific elongation.

Through the mechanical energy tests of tensile samples before and after corrosion, the mechanical properties of the C110-1 and C110-2 samples were compared with each other, as shown in Figure 16. It can be seen that the yield strength, tensile strength, and fractured elongation of C110-1 and C110-2 all decreased after corrosion, indicating that the mechanical properties of the materials were damaged by corrosion. In the liquid phase, the damage to yield strength, tensile strength, and fractured elongation of the two pipes was more serious than that in the gas phase. C110-2 material, mainly composed of needle ferrite, has lower yield strength due to its higher-density dislocation inside and more intense hydrogen traps from dispersed carbides than C110-1. In addition, various mechanical indices of the two corroded samples are very close, with only a slight difference, while the mechanical functions of C110-2 after corrosion turned out to be slightly superior to those of C110-1.

### 3.4. Analysis of Fracture Morphology

**Figure 17:**
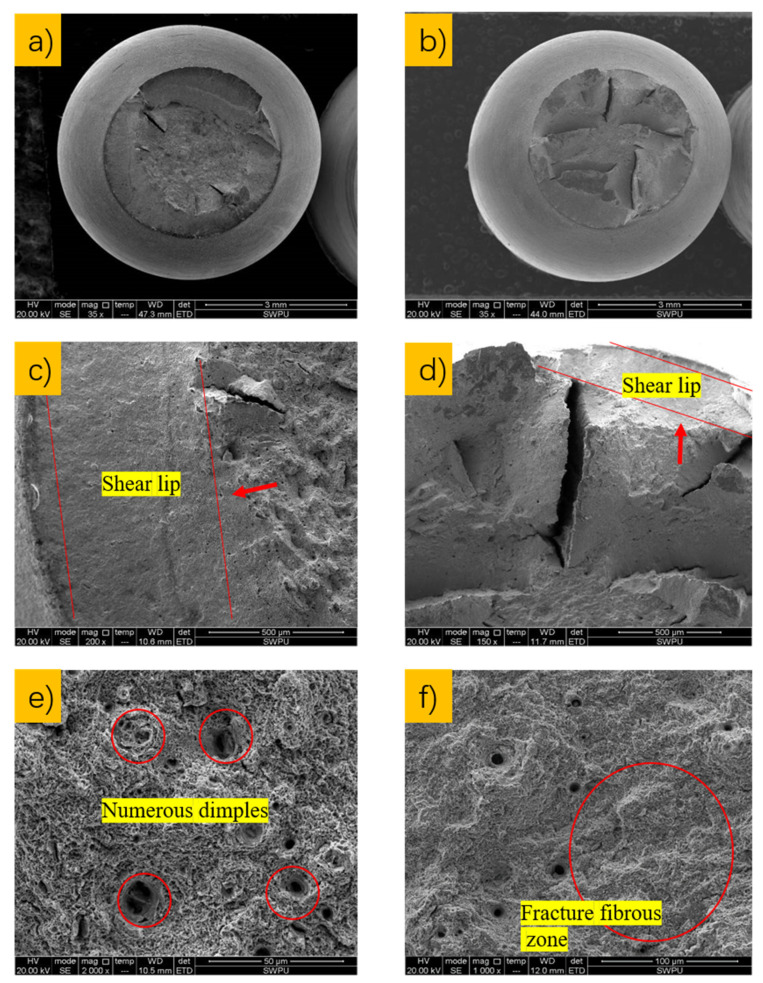
Tensile fracture morphology of the two steels in air at room temperature (25 °C): (**a**) C110-1 fracture with obvious shear lip and fibrous zone; (**b**) C110-2 ductile fracture with many secondary cracks; (**c**) C110-1 fracture with bigger shear lip; (**d**) C110-2 fracture with smaller shear lip; (**e**) C110-1 fracture fibrous zone of numerous dimples; (**f**) C110-2 fracture fibers.

**Figure 18:**
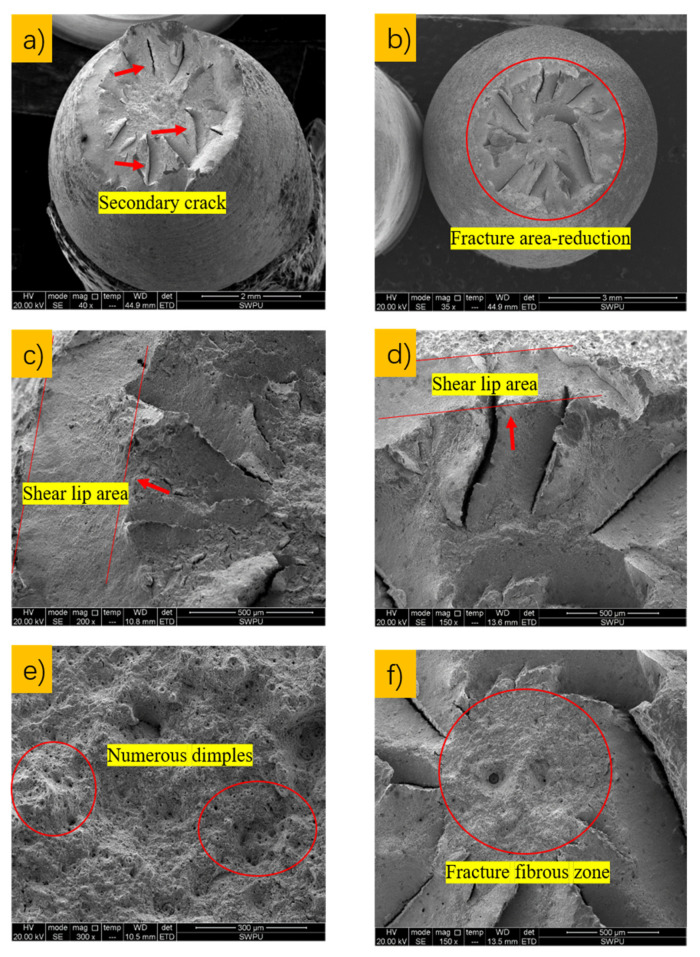
Tensile fracture morphology after stress corrosion with constant load in liquid phase: (**a**) C110-1 ductile fracture with secondary crack; (**b**) C110-2 ductile fracture with a high area-reduction rate; (**c**) C110-1 fracture with bigger area of the shear lip; (**d**) C110-2 fracture with smaller area of the shear lip; (**e**) C110-1 steel fracture with fibrous zone of many dimples; (**f**) C110-2 fracture with fibrous zone.

The tensile fracture surfaces after gas-phase and liquid-phase constant-load tests on C110-1 and C110-2 were analyzed by scanning electron microscopy (SEM), with the results shown in Figure 17 and Figure 18.

Through analysis, it was found that there were a certain number of dimples as ductile fractures on the surfaces of both the C110-1 and C110-2 samples after high-temperature, high-pressure stress corrosion testing with constant load. Comparing the fracture surfaces of the two samples in gas-phase medium, it can be seen that the shear lips in the micro-region of C110-1 sample are larger, and the diameter of the fibrous ductile fracture zone in C110-1 is smaller than that in C110-2. Therefore, it can be judged that C110-2 has better fracture resistance. On the other hand, it can be seen from Figure 18 that the necking shrinkage of C110-1 decreased significantly with expanded shear lips, and the fibrous ductile zone of the fracture surface also decreased obviously right after the high-temperature, high-pressure stress corrosion tests with constant load in liquid phase of the two samples. Although the C110-2 fracture after the test exhibited similar properties, its size reduction of the fibrous ductile area on fracture surfaces was smaller, basically maintaining the same characteristics as those of the gas-phase tensile fracture. It had a large fibrous zone area on the fracture surface, which could effectively delay the fracture of the sample. This illustrated that C110-2 has stronger resistance to environmental cracking and maintained a high level of ductility under the stress corrosion circumstances.

### 3.5. Analysis of Surface Damage

**Figure 19:**
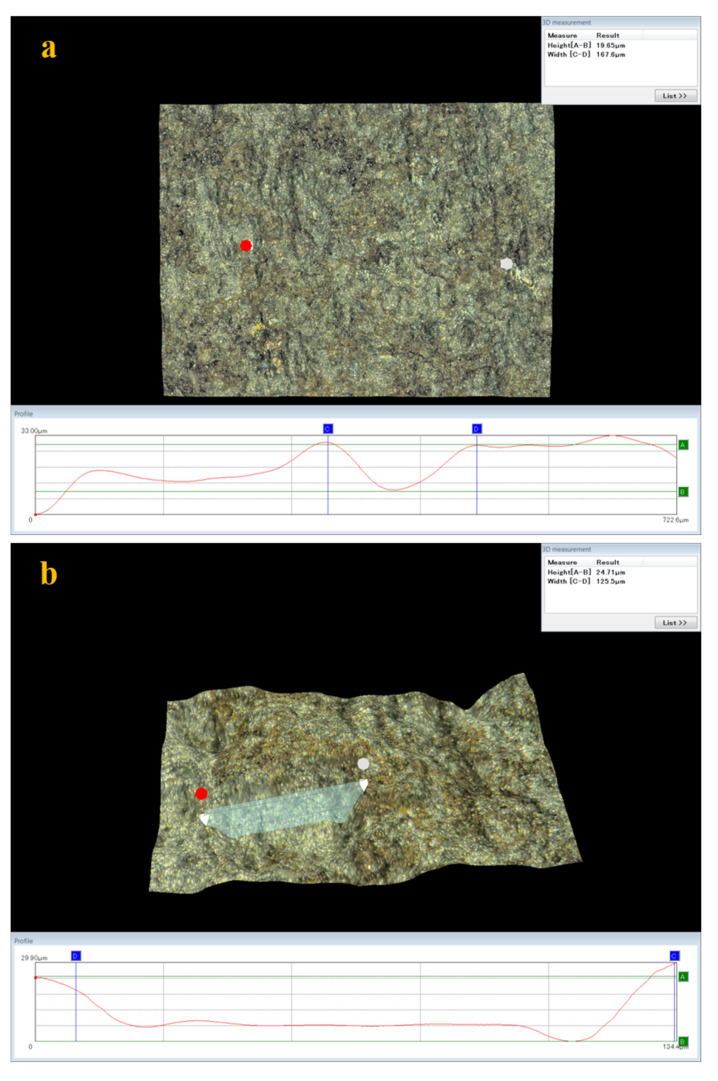

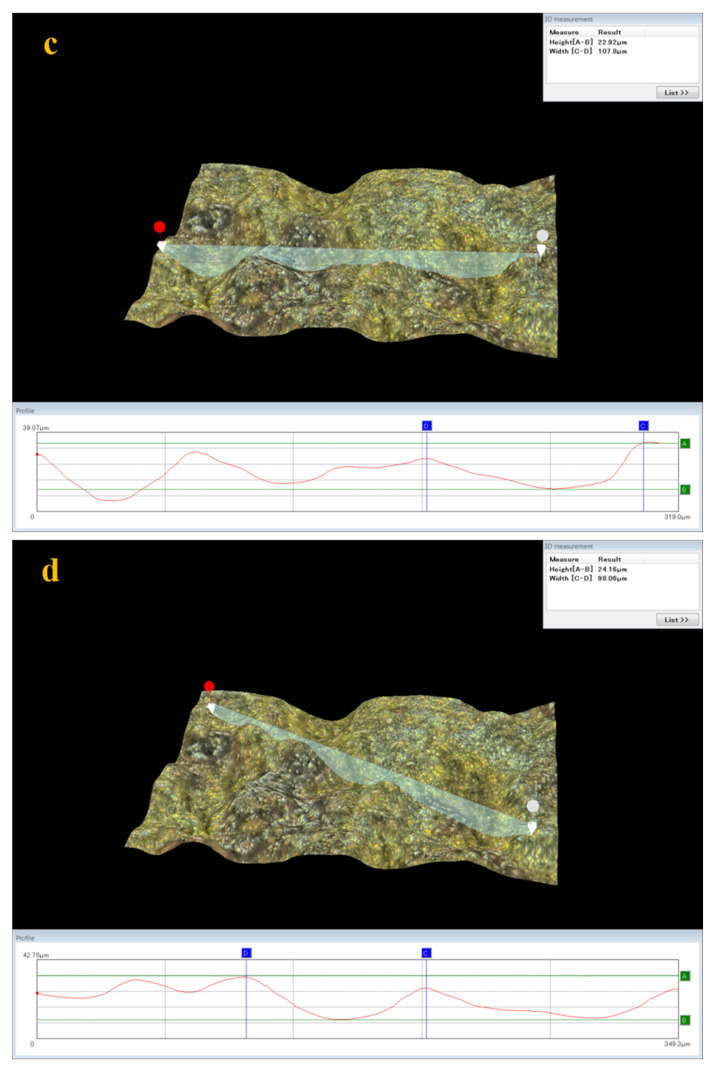

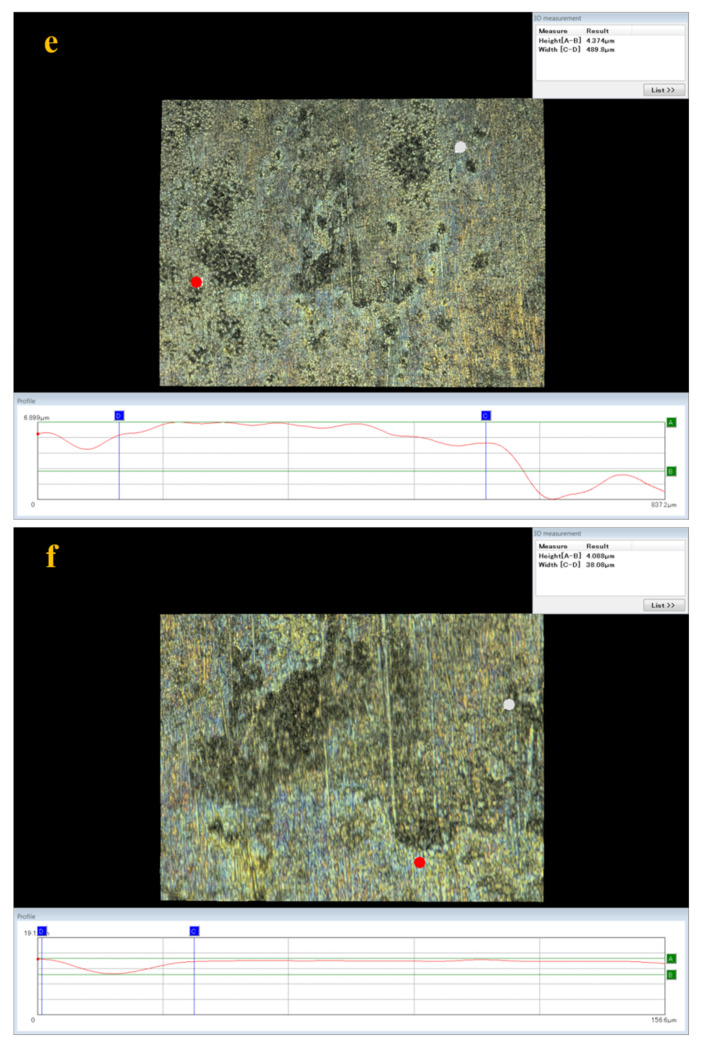

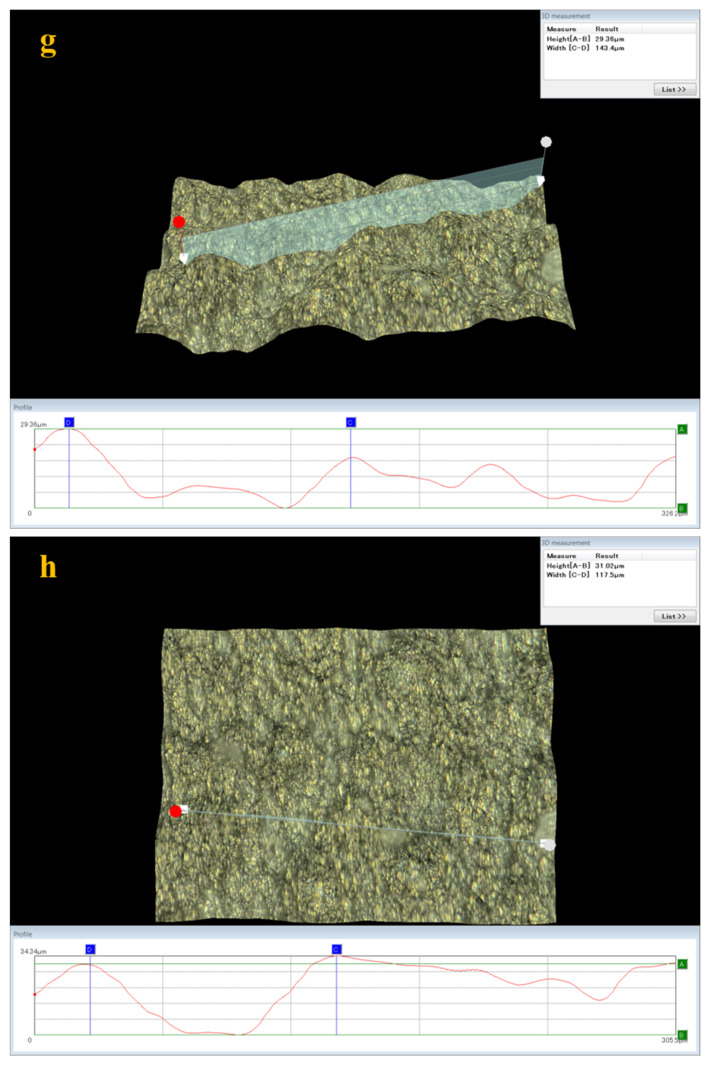
Sizes of pits: (**a**,**b**) C110-1 in gas; (**c**,**d**) C110-1 in liquid; (**e**,**f**) C110-2 in gas; (**g**,**h**) C110-2 in liquid.

**Figure 20:**
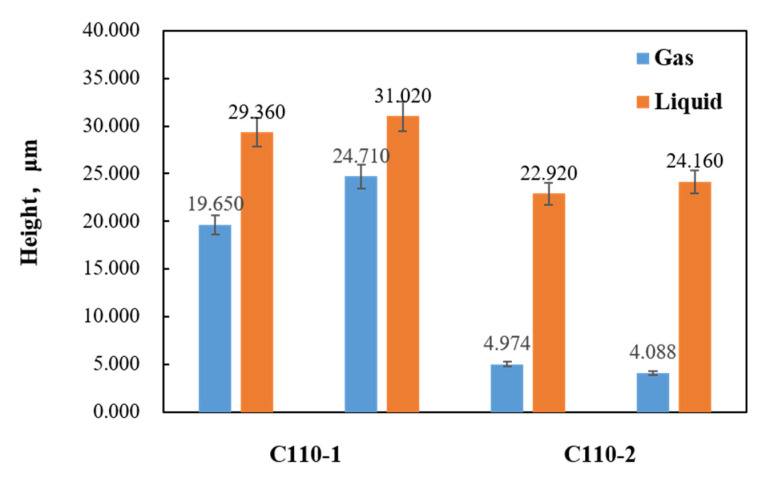
Height of pits.

Some studies in the literature [35,36] have pointed out that there is a strong relation between stress corrosion cracking and pitting corrosion, making it necessary to further study the pits on the surfaces of stress corrosion tensile samples with constant load under high temperature and high pressure. Two representative zones were selected on the sample surfaces where the morphology of the corrosion pits was analyzed and measured by ultra-depth-of-field 3D microscopy. It was found that some pits appeared on the sample surfaces after stress corrosion acting with constant load under high temperature and high pressure, as shown in Figure 19. Moreover, Figure 20 exhibits that pit depths on the surfaces of C110-1 and C110-2 samples after corrosion in a liquid-phase stress environment with constant load under high temperature and high pressure were greater than those in gas-phase medium. In identical corrosion medium, the depth of pits on the surface of the C110-1 sample after stress corrosion was greater than that on the C110-2 sample, indicating that these pits on C110-1 are more serious. As far as all are aware, pitting corrosion is one of the major causes of stress corrosion cracking in materials. Cracks often initiate at the pit with maximum stress concentration. The stress concentration at each pit is related to its depth, size, and shape, closely relevant to the stress state of the material.

**Figure 21:**
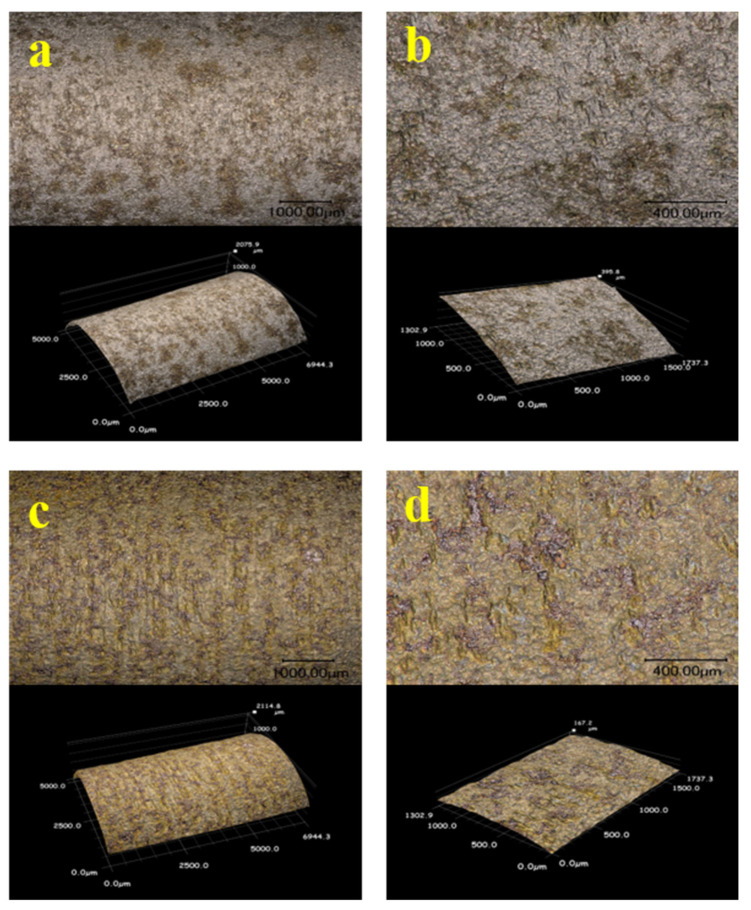
Passive film: (**a**) C110-1, 1000 μm; (**b**) C110-1, 400 μm; (**c**) C110-2, 1000 μm; (**d**) C110-2, 400 μm.

Furthermore, the passive film on the material surface was found to have an excellent inhibitory effect on the formation of pits. However, in the process of stress loading, the passive film is quite susceptible to damage and falloff, causing more severe pitting corrosion in the liquid phase. Therefore, it was imperative to observe the passive film on the sample surface after stress corrosion cracking testing with constant load under high temperature and high pressure in the liquid medium. We examined the passive film on the sample surface using a 3D microscope, as shown in Figure 21. It can be seen that there was still a small amount of dense passive film left on the surface of the C110-2 sample after the constant-load test under high temperature and high pressure; on the contrary, there was almost no passive film left on the surface of C110-1 to prevent pitting corrosion from further occurring, which is another reason why the stress corrosive cracking resistance of C110-1 is actually inferior to that of C110-2.

### 3.6. Effect of Hydrogen on Stress Corrosive Cracking

**Figure 22:**
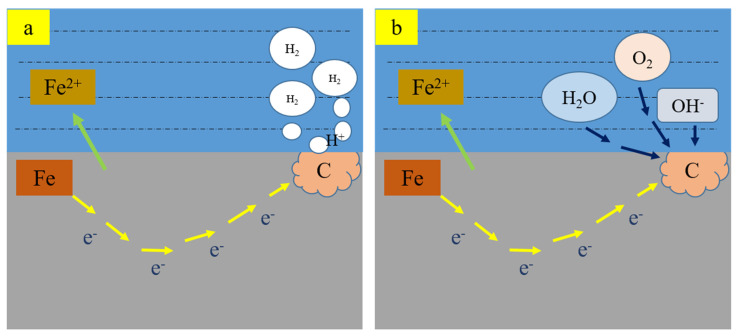
Mechanism of stress corrosive cracking: (**a**) Hydrogen evolution reaction; (**b**) Oxygen-absorption reaction.

Stress corrosive cracking of sulfide can be divided into the anodic dissolution type and the hydrogen-induced cracking type, according to their formation mechanisms. As shown in Figure 22, it is a hydrogen evolution reaction if the cathodic process corresponds to anodic metal dissolution (corrosion) (Equation (3)) where atomic hydrogen energy can diffuse into the sample to control crack nucleation and propagation; this kind of cracking is then called the hydrogen-induced cracking type of stress corrosion. On the other hand, it is an oxygen-absorption reaction (Equation (4)) if the cathodic process corresponds to anodic dissolution in the stress corrosion system, or the hydrogen infiltrating the sample is insufficient to cause hydrogen-induced cracking despite the cathode undergoing a hydrogen evolution reaction; then the crack nucleation and propagation caused by stress corrosion are controlled by the anodic dissolution process of metal, which is called the anodic-dissolution type of stress corrosion.

Electrochemical equations of corrosion [37]:

(2) $$\mathrm{Anode}:\;M\to M^{+n}+ne$$

(3) $$\mathrm{Cathode}:\;2H^{+}+2e^{-}\to H_{2}$$

(4) $$\mathrm{Or}:\;2H_{2}O+O_{2}+4e^{-}\to 4OH^{-}$$

In this paper, we studied the corrosion of materials under the synergistic action of stress, hydrogen, and corrosive media, including two meanings: one is the stress corrosion of hydrogen-induced cracking, in which the hydrogen comes from the stress corrosion process (cathodic hydrogen evolution); the other is the promotion of anodic dissolution stress corrosion by hydrogen infiltrating the sample in the stress corrosion process. Because stress corrosion may form pits on the surface through partial anodic dissolution, it can be regarded as a microcrack. For crackless specimens, the formation of pits plays a crucial role in stress corrosion, as there will be a stress concentration at the front end of the pits (microcracks); on the other hand, the interior of the corrosion pit will be partially acidified due to the occluded cell effect, which facilitates the hydrogen evolution reaction, leading to hydrogen infiltrating the sample to expand the corrosion pit and result in hydrogen-induced fracture.

The effect of H on the passive film can increase the vacancy. The existence of vacancy in the passive film promotes the diffusion of metal ions from the inner boundary of the film to the solution, resulting in the partial rupture of the film. Another major reason for this is that H can change the structure and properties of the passive film, which facilitates dissolved stress corrosion. Generally, hydrogen cannot infiltrate the oil casing pipe steel in a molecular state, but it enters into a certain position of the steel through a series of processes like physical adsorption, chemical adsorption, dissolution, and diffusion on the surfaces of the oil casing pipe steel.

Previous studies [38] have demonstrated that the outer electrons of hydrogen and the metal jointly form a conduction band after hydrogen penetrates into the metal, turning hydrogen into a positive ion. Normally, hydrogen exists in hydride in the form of negative ions, but when atomic hydrogen or ionic hydrogen come together, hydrogen molecules will be formed and exist in the form of hydrogen gas in the casing pipe. Hydrogen in the material is harmful, resulting in hydrogen damage; microcracks from hydrogen inclusion pressure; high-temperature, high-pressure hydrogen corrosion; martensitic transformation induced by the hydride phase or hydrogen itself; hydrogen-induced plastic loss; and hydrogen-induced cracking. Moreover, the action of stress will accelerate the infiltration of H into the metal material, expediting crack propagation and reducing *K*_ISSC_ value.

## 4. Conclusions

(1) Both C110-1 and C110-2 casing pipe materials meet the requirements of the ISO 11960 standard, but after the tests in DCB standard A solution, the two materials suffered a certain level of stress corrosion cracking and generated cracks. Therefore, it is essential to evaluate the environmental cracking applicability of these two C110 casing pipes by the high-temperature, high-pressure, constant-load method under circumstances simulating the actual working conditions.
(2) The carbides dispersed in the microstructure exist in the matrix in the form of hydrogen traps, providing many vacancies for the redistribution of infiltrated hydrogen atoms, which can effectively hinder the microregional hydrogen embrittlement caused by high hydrogen pressure in partial regions; this weakens the increase in the partial hydrogen concentration caused by the fusion of infiltrated hydrogen and strong hydrogen-traps in the material, and as a result, the possibility of crack generation is also reduced. Besides this, the dispersed carbide can improve the SSC resistance of the material as well.
(3) After stress corrosion cracking tests with constant load under high temperature and high pressure, both materials suffered a certain degree of stress damage. Between the two of them, the mechanical properties of C110-2 after corrosion were slightly better than those of C110-1.
(4) Through analysis of the tensile fracture, it was observed that C110-2 has stronger resistance to environmental cracking under stress corrosion circumstances and is able to maintain high ductility even after stress corrosion.
(5) Under the action of stress, the infiltration of H into the metal material is accelerated, expediting crack propagation and lowering the *K*_ISSC_ value.

## Figures and Tables

**Table 1:** Chemical compositions of C110-1 and C110-2 steels (wt.%).

Steel	C	Si	Mn	P	S	Ni	Cr	Cu	Ti	Nb	Mo	V
C110-1	0.25	0.21	0.50	0.007	0.001	0.03	0.54	0.03	0.05	0.04	0.65	0.11
C110-2	0.27	0.27	0.51	0.008	0.001	0.03	1.09	0.01	0.02	0.04	0.56	0.10
ISO C110	Max 0.35	-	Max 1.2	Max 0.020	Max 0.003	Max 0.99	0.4–1.5	-	-	-	0.25–1.0	-

**Table 2:** Mechanical properties of C110-1 and C110-2.

Steel	Yield Strength, MPa	Tensile Strength, MPa	Elastic Modulus, GPa	Yield Ratio	Elongation after Fracture, %
C110-1	833 (120 ksi)	887 (128 ksi)	210	0.94	20.8
C110-2	837 (121 ksi)	908 (131 ksi)	220	0.92	24.5

**Table 3:** Cantilever displacement of oil casing pipes.

Level ^(A)^	Yield Strength Range	Approved Cantilever Displacement (*δ*)
	MPa	mm
L-80	552–655	0.71–0.97
C-90	621–724	0.64–0.89
C-95,T-95	655–758	0.58–0.84
Level 100	689–793	0.51–0.76
Level 105	724–827	0.46–0.71
Level 110	758–862	0.38–0.64
P-110	758–965	0.25–0.64
Q-125	862–1030	0.25–0.51

^(**A**)^ ISO 11960 rating unless otherwise specified.

**Table 4:** Ion contents and total salinity of the simulated formation water.

Main Content Types	Main Ion Content, g/L							Density, g/cm^3^	pH Value	Total Salinity, g/L
	Na^+^	K^+^	Ca^2+^	Mg^2+^	Cl^−^	SO_4_^2−^	HCO_3_^−^			
Contents	59.00	10.04	11.43	2.43	126.52	0.71	0.71	0.996	5.38	210.84

**Table 5:** Dosages of reagents used in preparing the simulated formation water.

Reagent Name	Molecular Weight, g/mol	Dosage, g/L
NaCl	58.44	148.37
KCl	74.55	19.20
MgCl_2_·6H_2_O	203.20	20.35
CaCl_2_	110.98	31.70
NaHCO_3_	83.99	0.98
Na_2_SO_4_	142.04	1.05

**Table 6:** Test results of fracture ductility: *K*_ISSC_ values of C110-1 and C110-2.

Number	Material	*P*, N	*a*, mm	*h*, mm	*B*, mm	*Bn*, mm	*K*_ISSC_, MPa∙m^1/2^	
							Tested	Average
1	C110-1	1510	46.78	12.70	9.51	6.25	27.18	27.16
2		1660	42.79	12.70	9.53	6.25	27.71	
3		1450	47.66	12.70	9.52	6.25	26.58	
4	C110-2	1510	46.98	12.70	9.52	6.25	27.27	28.19
5		1500	50.90	12.70	9.52	6.25	29.03	
6		1540	47.89	12.70	9.51	6.25	28.28	
